# Hydration and its Hydrogen Bonding State on a Protein Surface in the Crystalline State as Revealed by Molecular Dynamics Simulation

**DOI:** 10.3389/fchem.2021.738077

**Published:** 2021-10-18

**Authors:** Hiroshi Nakagawa, Taro Tamada

**Affiliations:** ^1^ Materials Science Research Center, Japan Atomic Energy Agency, Ibaraki, Japan; ^2^ J-PARC Center, Japan Atomic Energy Agency, Ibaraki, Japan; ^3^ Institute for Quantum Life Science, National Institutes for Quantum Science and Technology, Ibaraki, Japan

**Keywords:** protein hydration, hydrogen bond, protein crystal, molecular dynamics simulation, neutron crystallography

## Abstract

Protein hydration is crucial for the stability and molecular recognition of a protein. Water molecules form a hydration water network on a protein surface via hydrogen bonds. This study examined the hydration structure and hydrogen bonding state of a protein, staphylococcal nuclease, at various hydration levels in its crystalline state by all-atom molecular dynamics (MD) simulation. Hydrophilic residues were more hydrated than hydrophobic residues. As the water content increases, both types of residues were uniformly more hydrated. The number of hydrogen bonds per single water asymptotically approaches 4, the same as bulk water. The distances and angles of hydrogen bonds in hydration water in the protein crystal were almost the same as those in the tetrahedral structure of bulk water regardless of the hydration level. The hydrogen bond structure of hydration water observed by MD simulations of the protein crystalline state was compared to the Hydrogen and Hydration Database for Biomolecule from experimental protein crystals.

## Introduction

In an aqueous environment, there is hydration water on a protein surface (Nakasako et al., 2004; Niimura et al., 2006; Gnesi et al., 2017). Hydration water plays an important role in the structural dynamics, stability, and functional expression of a protein (Rupley et al., 1983; Careri et al., 1998). Hydration water not only forms hydrogen bonds with amino acid residues in proteins but also between water molecules in the hydration layers. As a result, a hydration water network is formed on the protein surface (Nakasako et al., 2004). The protein hydration structure has been studied by crystallography. Many hydration water molecules are observed at cryogenic temperatures by X-ray crystallography (Nakasako et al., 1999). Hydration structures are efficiently observed at room temperature by neutron crystallography (Niimura et al., 2006). The structure and dynamics of hydration water are examined in protein crystals (Podjarny et al., 1997). Based on crystal structures, including hydrogen atoms in the Protein Data Bank (PDB), the Hydrogen and Hydration Database for Biomolecules (HHDB ^©^ National Institutes for Quantum and Radiological Science and Technology (QST) licensed under CC BY-SA4.0 International; doi: 10.18908/lsdba.nbdc00495-000.V002) has been constructed (Niimura et al., 2006). In this database, hydration structures and hydrogen bonding states observed in the crystal structure analysis of biological macromolecules are summarized.

Protein hydration has also been analyzed by molecular dynamics (MD) simulations (Higo et al., 2002). In this MD simulation in solution, the solvent density and solvent dipole on the protein surface were in good agreement with the hydration structure observed by crystal structure analysis, indicating that the combination of computational and experimental analysis is effective in protein hydration studies. Most MD simulations of proteins are performed in solution, but the protein’s conformation, dynamics, and function are changed in the crystalline state from in a solution (Li et al., 2017; Srivastava et al., 2018; Konold et al., 2020). Because the limitation of crystallography in not analyzing the protein dynamics is discussed (Srivastava et al., 2018), MD simulations in a protein crystalline state should be useful to compare them to experimental protein crystallography. Many MD simulations of protein crystalline states have been performed (Meinhold et al., 2005; Hu et al., 2008; Joti et al., 2008; Janowski et al., 2016; Cerutti et al., 2018). It should be also effective to quantitatively compare the experimental and calculated protein and hydration structures in crystals to evaluate the validity of MD simulations. In some experiments on protein dynamics, such as inelastic and quasi-elastic neutron scatterings, dielectric relaxation, and terahertz spectroscopy, hydrated powder proteins are used as samples (Nakagawa et al., 2010; Yamamoto et al., 2021). MD simulations of protein dynamics in the crystalline state have been shown to quantitatively reproduce neutron quasi-elastic scattering spectra of hydration water as well as protein (Tarek et al., 1999; Tarek et al., 2000). The structure and dynamics of biomolecules and hydration water in the crystalline state will also provide useful information for various spectroscopic experiments.

Enzyme activity decreases with the decrease in hydration level (Careri et al., 1998). With decreasing water content, the molecular mobility of both protein and hydration water decreases, and proteins become vitrified. Minimum hydration is necessary for enzymatic activity (Careri et al., 1998), and its threshold hydration level is correlated to percolation transisiton of hydration water (Careri et al., 1998; Nakagawa et al., 2010). Protein crystallography and MD simulations have been performed under varying humidity conditions to investigate the protein conformation and hydration states at atomic resolution (Kodandapani et al., 1990; Hu et al., 2008; Takayama et al., 2011; Trampari et al., 2018; Salinas-Garcia et al., 2019).

In this study, MD simulations of staphylococcal nuclease (SNase) in the crystalline state were performed at various hydration levels. The hydration level-dependent hydration structure and hydrogen bonding states were analyzed. As the water content increased, amino acid residues on a protein surface were uniformly more hydrated, and the number of hydrogen bonds per single water asymptotically approached 4, the same as bulk water. The distances and angles of hydrogen bonds in hydration water in the protein crystal were almost identical to those in the tetrahedral structure of bulk water regardless of water content. The hydrogen bond structure of hydration water observed by MD simulations of the protein crystalline state was compared to the HHDB from the experimental protein crystal.

## Materials and Methods

### MD Simulation of Protein Crystal and Analysis of the Trajectories

**FIGURE 1:**
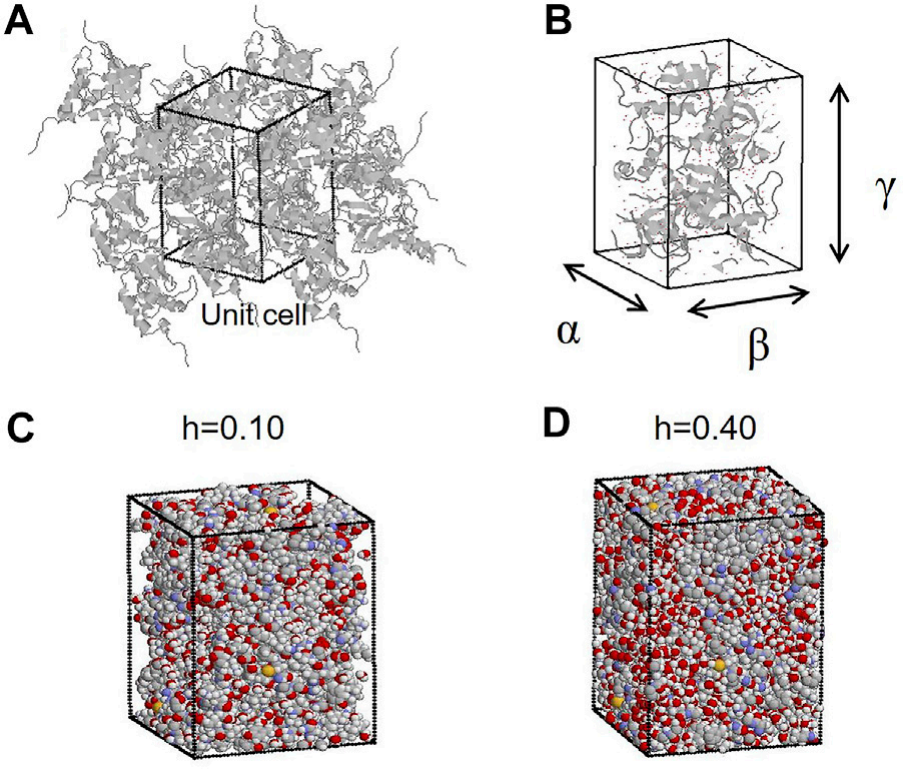
**(A)** MD simulation system of protein crystals (with periodic boundary condition) of SNase. The rectangular box indicates the unit cell. **(B)** Lengths of the three sides of a rectangular unit cell (lattice constants) are denoted by α, β, and γ. **(C** and **D)** Proteins and water molecules in the unit cell at hydration levels of h = 0.10 (g water/g protein) and h = 0.40 (g water/g protein). In this figure, all the atoms outside the unit cell are put inside the unit cell according to the periodic boundary condition.

The crystal structure of SNase (PDB code: 1EY0) was used as the initial simulation structure. The simulated system was constructed to reproduce the crystal unit cell, having a space group symmetry of *P*4_1_ (Figures 1A,B). Missing residues 1 to 5 and 142 to 149 in 1EY0 were modeled based on the nuclear magnetic resonance structures (PDB code: 1JOR). The system contained four protein molecules, including crystal water registered in 1EY0. In addition, water molecules were added randomly distributed in the empty space in each system (Figures 1C,D) to construct the protein crystal with different hydration levels (h) from 0.10 to 0.55 (g water/g protein). The number of water molecules and atoms in the simulation system are summarized in Supplementary Table S1. To neutralize the system, 32 chloride ions were randomly placed in the system. Apart from MD simulations of crystalline proteins, MD simulations of bulk water were also performed. For the simulation of bulk water, 440 water molecules were set in a rectangular box. The periodic boundary condition was imposed, and the particle mesh Ewald method was used with a cutoff of 10 Å. The AMBER *ff14SB* force field and the TIP3P water model were employed. After energy minimization (2000 steps), 2 ns MDs were performed to equilibrate the systems at 300 K and 1 atm, which were maintained following the Berendsen method (relaxation time of 1 ps for both) using the program AMBER (Case et al., 2015). For equilibration MD, the ensemble is NPT. Successive 4 ns trajectories in the NPT ensemble were obtained for the analyses. For hydrogen bond analysis, pairs of water molecules were selected as hydrogen-bonded only if their interoxygen distance was <3.5 Å and simultaneously the O–H…O angle was > from 120° to 180°. To check the reproducibility of the simulation results, several simulations from different initial atomic velocities from a Boltzmann distribution were performed. The analytical results did not depend on the trajectories of different times and initial states. The thermal equilibration of the simulation was checked through total energy in the system and the temperature. Supplementary Figure S1 shows the lattice constants and simulation boxes as a function of the hydration level. As the hydration level increases, these parameters increase to keep a constant pressure in the system. As for the distance between proteins in an unit cell, the contact maps between proteins were calculated (Joti et al., 2008). At low water content, there is more protein-protein contact, and at high water content, hydration water enters the gaps between the proteins, resulting in less protein-protein contact.

## Results and Discussion

### Hydration Structure and Distribution of Water Molecules on the Protein Surface

**FIGURE 2:**
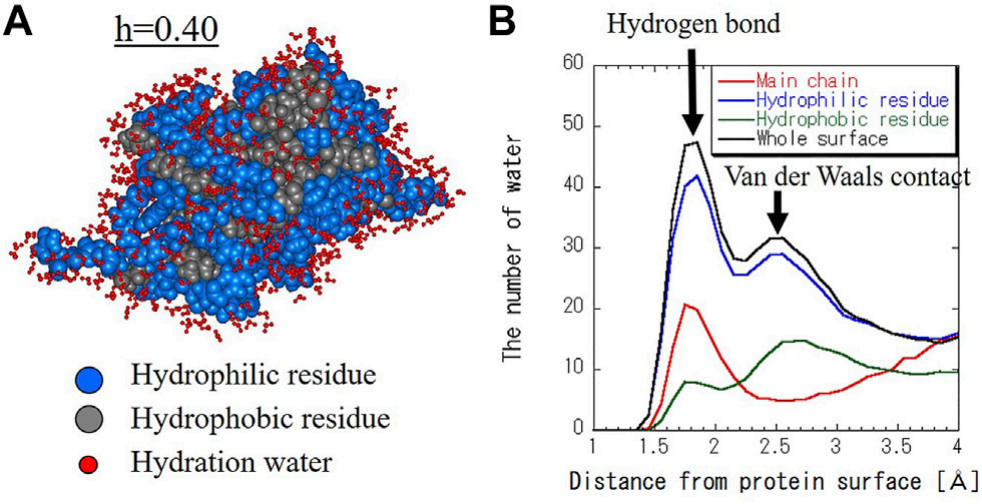
**(A)** Structure of hydrated SNase at h = 0.40 (g water/g protein). Hydrophilic and hydrophobic residues are blue and gray balls, respectively. Hydration water molecules whose oxygen atom is within 4 Å from the protein surface are shown in red. **(B)** The number of hydration water as a function of distance from the protein surface for the main chain, hydrophilic and hydrophobic residues, and whole surface. The distance between the protein surface and the water molecules was calculated as the distance between the atoms that make up the protein and the oxygen atoms of the water molecules.

Figure 2A shows the molecular structure and hydration water of SNase with the hydration level of h = 0.40 as a snapshot of the MD trajectory. Hydration water spreads not only on the hydrophilic surface but also on the hydrophobic surface. Figure 2B shows the number of hydration water around hydrophilic residues, hydrophobic residues, and the main chain per protein as a function of the distance from the protein surface. Hydration water around the main chain is mainly present at the hydrogen bond distance (Kumar et al., 2009). This indicates the water molecules that are hydrogen-bonded to the peptide bonds. For hydrophilic residues, the first major peak is at the hydrogen bond distance and the second peak is at the van der Waals (VDW) distance. For hydrophobic residues, there is more hydration at the VDW distance than the hydrogen bond distance. This is a reasonable result because hydrophobic surfaces have fewer donors and acceptors for hydrogen bonding.

**FIGURE 3:**
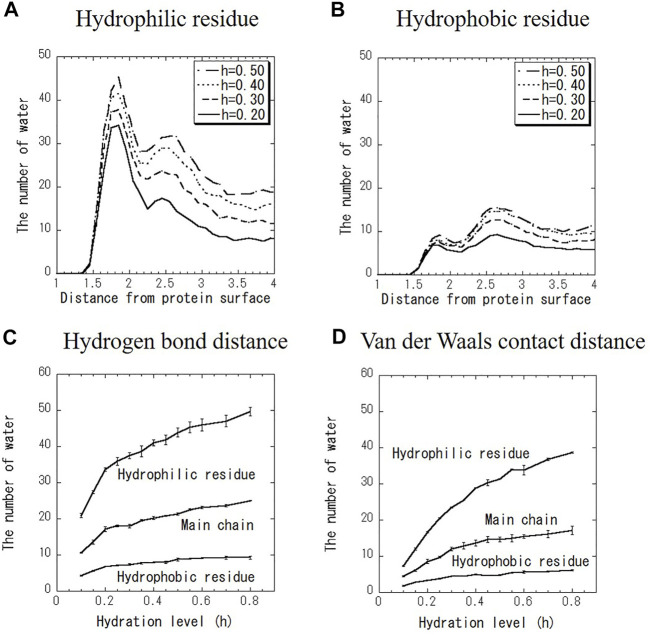
Distribution of the number of water molecules on (A) hydrophilic residue and (B) hydrophobic residue. Hydration level dependence of the number of water molecules at (C) hydrogen bond distance (1.8 Å) and (D) VDW contact distance (2.5 Å) for hydrophilic and hydrophobic residues and the main chain.

Figures 3A,B shows the distribution of the number of water molecules on the hydrophilic and hydrophobic residues at various hydration levels. Data at every hydration level are shown in Supplementary Figure S2. As shown in Figures 3A,B, the number of hydration water on hydrophilic and hydrophobic surfaces has the one peak at the hydrogen bond distance and another peak at the VDW distance at every hydration level. The former peak is more intense for hydrophilic surface, and vice versa for hydrophobic one. For example, hydration water molecules around hydrophilic residues are hydrogen-bonded to carboxyl groups and amino groups of amino acid side chains. On the hydrophobic surface, hydrated water with pentagonal (five-membered) ring structures were observed (See Supplementary Figure S3). Such characteristic hydration structures have been previously confirmed by crystal structure analysis and MD calculations (Teeter et al., 1984; Lounnas et al., 1994; Nakasako et al., 2004). The shapes of the distributions in Figures 3A,B change similarly to hydration level. Figures 3C,D shows the number of hydration water at the two peaks of the hydrogen bond distance and VDW distance around hydrophilic residue, hydrophobic one and the main chain as a function of hydration level. The magnitudes of both peaks increase with the increase in hydration level. With increasing hydration levels, the number of hydration water does not increase preferentially around hydrophilic residues but increases uniformly around hydrophobic residues as well. This result suggests that hydration water is spread evenly over the entire protein surface rather than aggregating on the hydrophilic protein surface.

**FIGURE 4:**
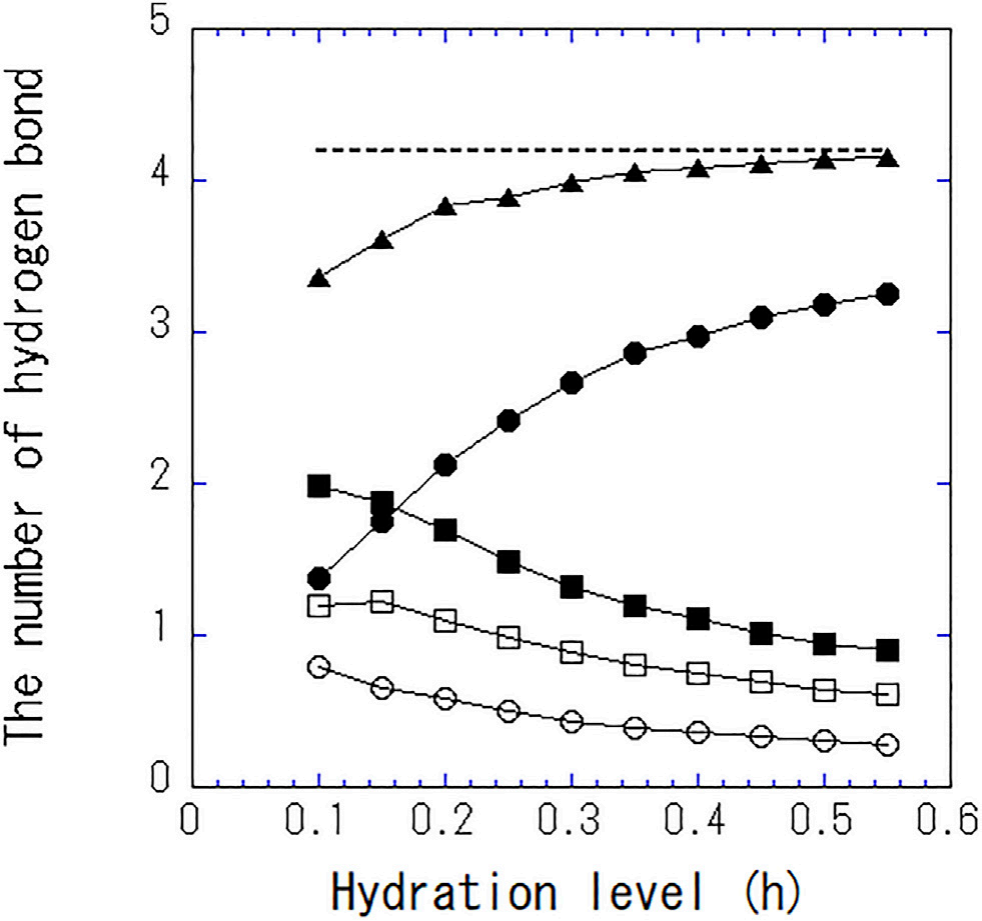
The number of total hydrogen bonds per single water (▲), between water and water (●), between water and protein (■), between water and the side chain of the amino acid (□), and between water and the main chain of the amino acid (○). The dashed line indicates the number of hydrogen bond per single water in bulk water.

Figure 4 shows the number of hydrogen bonds per one water molecule at different hydration levels. As hydration level increases, the number of hydrogen bonds between water and water increases, whereas that between water and protein decreases. The total number of hydrogen bonds per single water asymptotically approaches 4, the same as bulk water. The hydrogen bonding with protein is replaced with that with water. At high hydration levels, approximately one of the four hydrogen bonds in a single water is bound to the protein and three are bound between water molecules.

### Geometry of the Hydrogen Bond in Hydration Water

**FIGURE 5:**
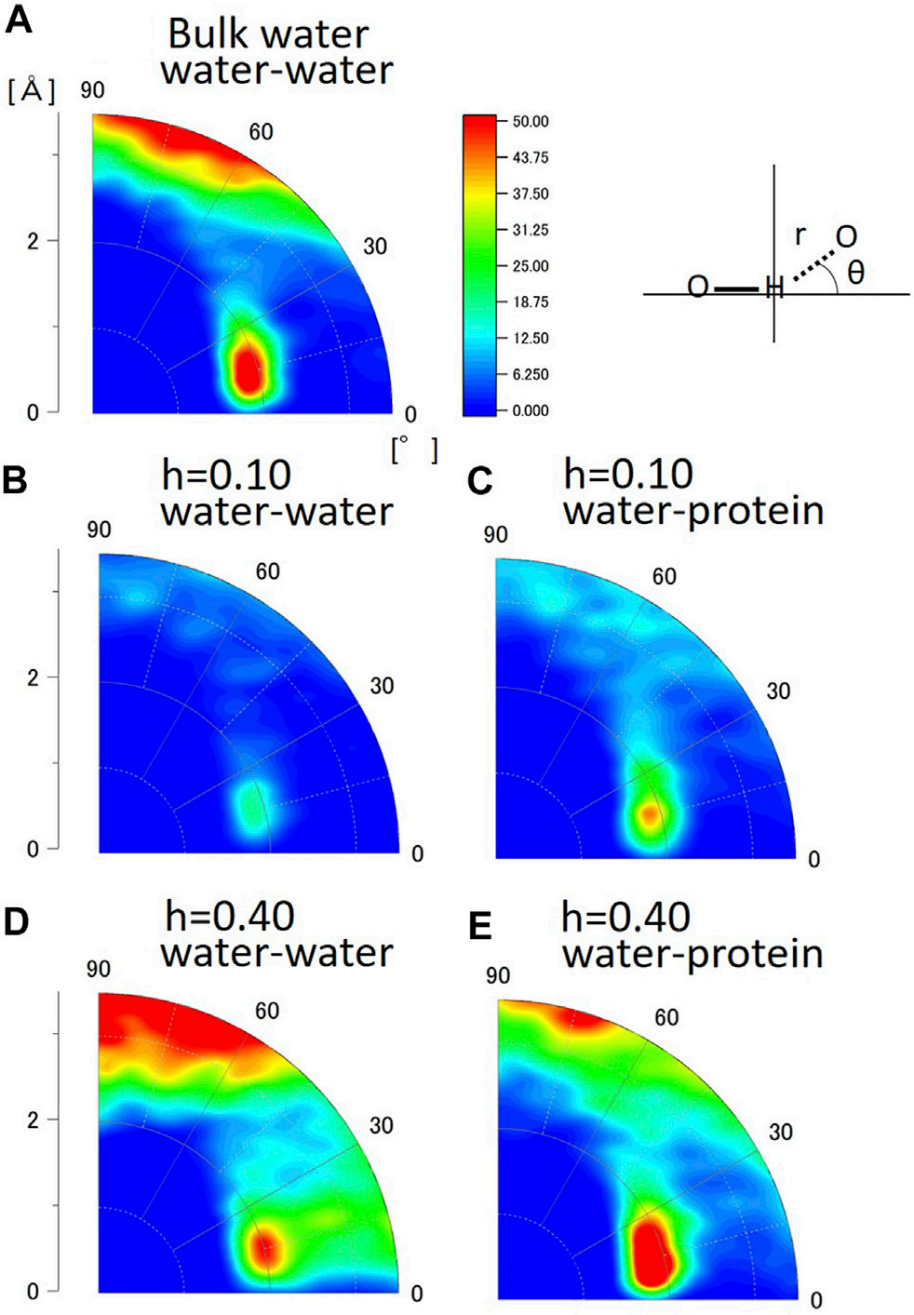
Geometrical distribution of O-H…O calculated from MD simulation **(A)** between water and water in bulk water, **(B)** water and water at h = 0.10 (g water/g protein), **(C)** water and protein at h = 0.10 (g water/g protein), **(D)** water and water at h = 0.40 (g water/g protein), and **(E)** water and protein at h = 0.40 (g water/g protein).

Next, the hydrogen bond structures of water molecules were analyzed in a two-dimensional map of distance and angle. Figure 5 shows the geometrical distribution of O-H…O between water and water in bulk water, between water and water, and between water and protein in the hydrated crystalline protein at h = 0.10 and h = 0.40. The distribution data for all hydration levels from h = 0.10 to h = 0.55 are shown in Supplementary Figure S4. The angle of O-H…O is 180°-θ (see the sketch in Figure 5). The peak in the distribution of O-H…O between water and water in bulk water is located at r = 2.0Å and θ = 15°. This geometrical feature suggests the tetrahedral hydrogen bond structure of water, as shown in Supplementary Figure S5. Crystallographic studies of proteins at low temperatures have also shown the presence of similar hydration water-formed hydrogen-bonded tetrahedral structures, suggesting their contribution to protein stability and folding (Nakasako, 2004). The present analysis shows that the tetrahedral structure of hydration water on the protein surface is independent of hydration level. The distribution of r > 3.0 Å at 60° < θ < 90° in bulk water indicates the presence of water molecules in proximity, but they do not form hydrogen bonds at this geometric location. The peak in the distributions were also observed for the geometrical distribution of O-H…H of between water and water (Figures 5B,D) and between water and protein (Figures 5C,E) in the hydrated protein crystal. These results suggest that the hydrogen bond structure of hydration water is similar to that of bulk water, and that the O-H…O positional relationship between water molecules in the hydration layers and protein is also similar to that of bulk water. The hydrogen bonds of water molecules maintain their tetrahedral structure regardless of their interaction with proteins or in a dry environment.

### Hydrogen and Hydration in Biomolecules

**FIGURE 6:**
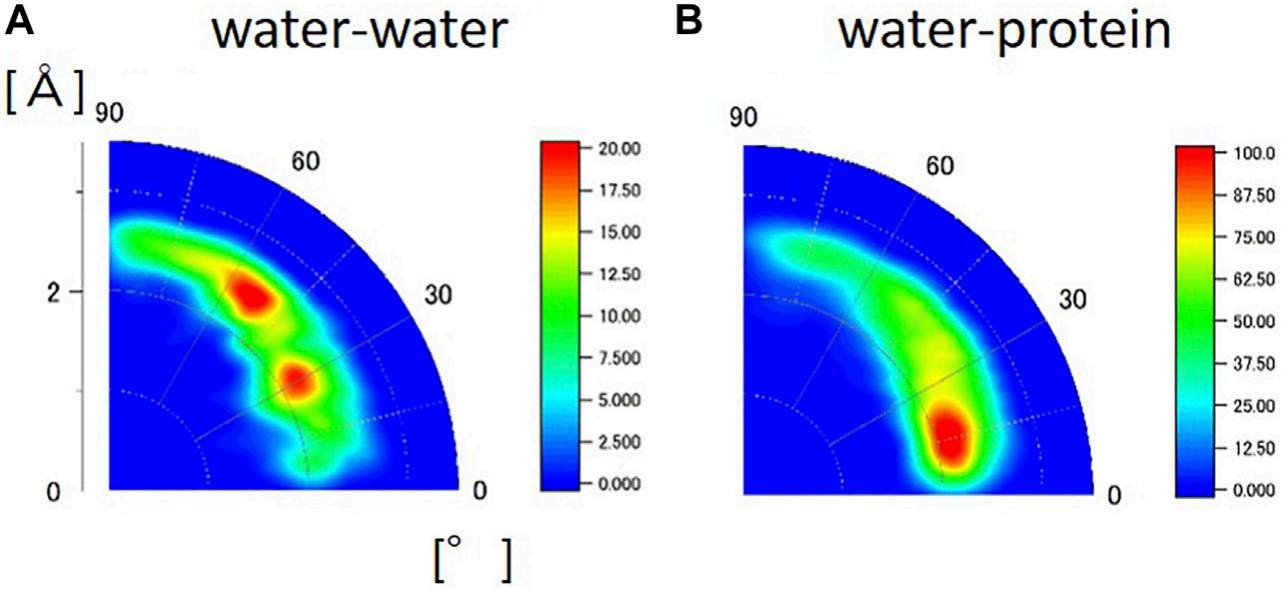
Geometrical distribution of O-H…O calculated from HHDB **(A)** between water and water and **(B)** water and protein from the HHDB. Neutron data above 2.0 Å resolution are used for the analysis.

The HHDB is a database of hydrogen atom positions in biological macromolecules, such as protein and DNA, and hydration water molecules, based on the selected PDB data (neutron crystallography and high-resolution X-ray crystallography). Figure 6 shows the geometrical distribution of O-H…O between water and water and between water and protein from the HHDB. Neutron data above 2.0 Å resolution are used for the analysis. In Figure 6A, the two peaks are observed in the geometrical distribution of O-H…O between water and water at the distance of around 2.0 Å and the angle of around 30° and 55°. The distance is equal to the distance obtained by MD simulation (Figures 5A,B,D). The angle of the peak position around 30° should correspond to that around 15° in the hydrated crystalline protein by MD simulations. Although the reason for the difference is unclear, it could be ascribed to the hydrogen bond in the tetrahedral geometry. The second peak around 55° is not observed in the hydrated crystalline protein (Figures 5B,D) or bulk water (Figure 5A) in the MD simulation. A typical case of the relative geometry of two water molecules observed at r = 2.5 Å and θ = 55° is shown in Supplementary Figure S6; (Tamada et al., 2009). In this geometry, a hydrogen bond can be formed. This arrangement may indicate some kind of hydration water cluster, although they do not necessarily form a tetrahedral structure. The water molecules registered in the HHDB may be water molecules that form hydrogen bonds in some cases; in other cases, water may be registered against the observed electron density or nuclear density regardless of hydrogen bond formation. This difference may lead to the discrepancy between HDDB and MD calculations. In the geometrical distribution of O-H…O between water and protein, as shown in Figure 6B, the peak in the distribution is located at r = 2.0 Å and θ = 15°. This is in good agreement with that in the hydrated protein crystalline state in MD simulation (Figures 5C,E). The geometrical relationship between water and protein in the HHDB is thought to maintain the tetrahedron of water molecules, consistent with low-temperature crystal structure analysis.

## Conclusions

MD simulations shows that, on the surface of hydrophilic residues, hydration water was abundant at the hydrogen bond distance from the protein surface. In contrast, on hydrophobic residues, hydration water was more abundant at the VDW distance than at the hydrogen bond distance. With increasing hydration levels, both types of residues were uniformly more hydrated, and the number of hydrogen bonds per single water asymptotically approached 4, the same as bulk water. The hydrogen bond structure of protein hydration water was shown to be independent of hydration level and adopt a tetrahedral structure like bulk water. The hydrogen bond between water and protein observed in the protein crystalline state in MD simulations is consistent with the hydrogen bond structure of hydrated water observed in neutron crystallography presented in the HHDB. In contrast, the hydrogen bonds between water and water are slightly different from each other. The difference could be the arbitrary nature of hydrogen bonds in water molecules registered in the HHDB. MD simulations of proteins in crystalline states should be useful for the combined analysis of protein hydration by crystallography, especially neutron crystallography, an effective tool for analyzing hydrogen and hydration structures.

## Data Availability

The original contributions presented in the study are included in the article/Supplementary Material, further inquiries can be directed to the corresponding author.
