# Supplementary material for: Hydration and its Hydrogen Bonding State on a Protein Surface in the Crystalline State as Revealed by Molecular Dynamics Simulation
Source: Front Chem. 2021 Oct 18;9:738077. doi: 10.3389/fchem.2021.738077 (PMC8558535; doi:10.3389/fchem.2021.738077)
Supplement: Supplementary file 1 [file DataSheet1.pdf]

Supplementary Materials

Supplementary Table S1. The number of water molecules and atoms in simulation systems at various hydration levels.

Hydration level (g water/g protein)	Water	Atoms
0.10	373	10.699
0.15	559	11.257
0.20	746	11.818
0.25	932	12.376
0.30	1110	12.910
0.35	1309	13.507
0.40	1494	14.062
0.45	1682	14.626
0.50	1867	15.181
0.55	2062	15.766

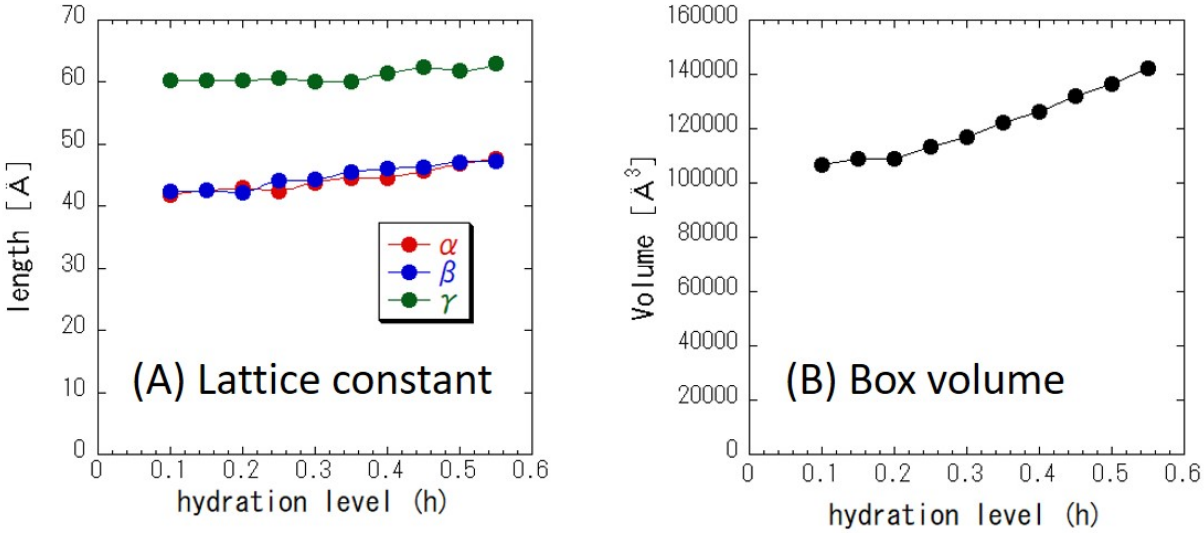

Supplementary Figure S2. (A) Lattice constants (α, β, and γ) and (B) box volume of unit cell in the MD simulation system of crystalline SNase at various hydration levels.

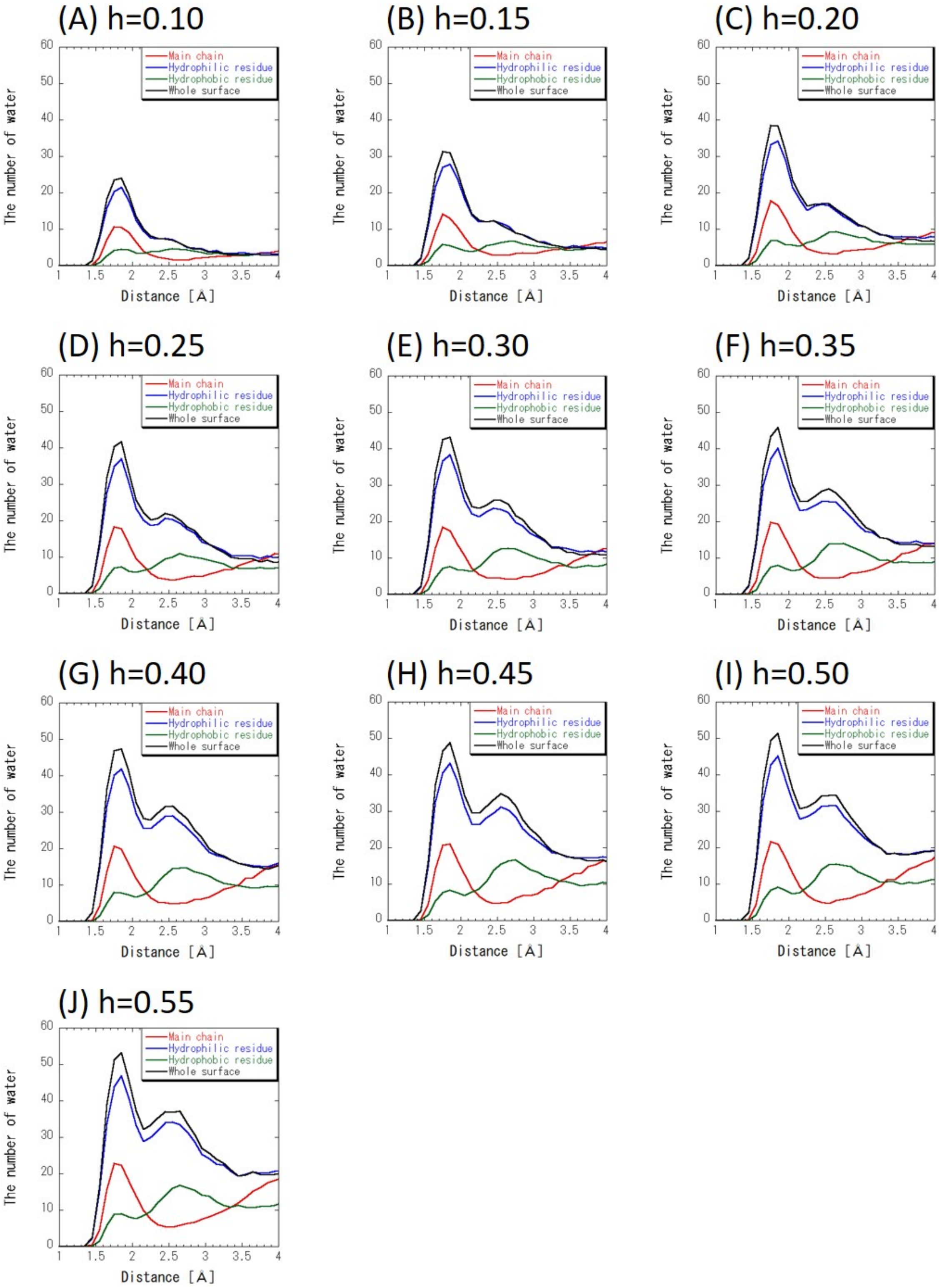

Supplementary Figure S2. The number of hydration water as a function of distance from the protein surface for the main chain, hydrophilic residue, hydrophobic one at various hydration levels, and whole surface.

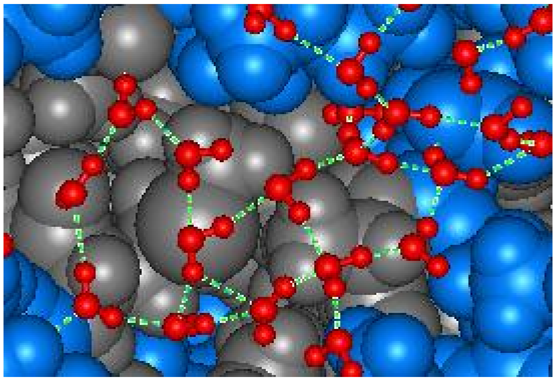

Supplementary Figure S3. On the hydrophobic surface, hydrated water with pentagonal (five-membered) ring structures were observed in our MD simulation. Such characteristic hydration structures have been previously confirmed by crystal structure analysis and MD calculations (Teeter, et al., 1984, Lounnas, et al., 1994, Nakasako et al., 2004).

References:

Teeter, M. M. (1984) Water structure of a hydrophobic protein at atomic resolution: pentagon rings of water molecules in crystals of crambin. *Proc. Natl Acad. Sci. USA* 81, 6014–6018. doi: 10.1073/pnas.81.19.6014

Lounnas, V., Pettit, M. (1994) A connected-cluster of hydration around myoglobin: correlation between molecular dynamics simulations and experiment. *Proteins* 18, 133-147. doi: 10.1002/prot.340180206

Nakasako, M. (2004). Water-protein interactions from high-resolution protein crystallography. *Phil. Trans. R. Soc. Lond. B* 359, 1191-1206. doi: 10.1098/rstb.2004.1498

(A) Hydrogen bond between water-water

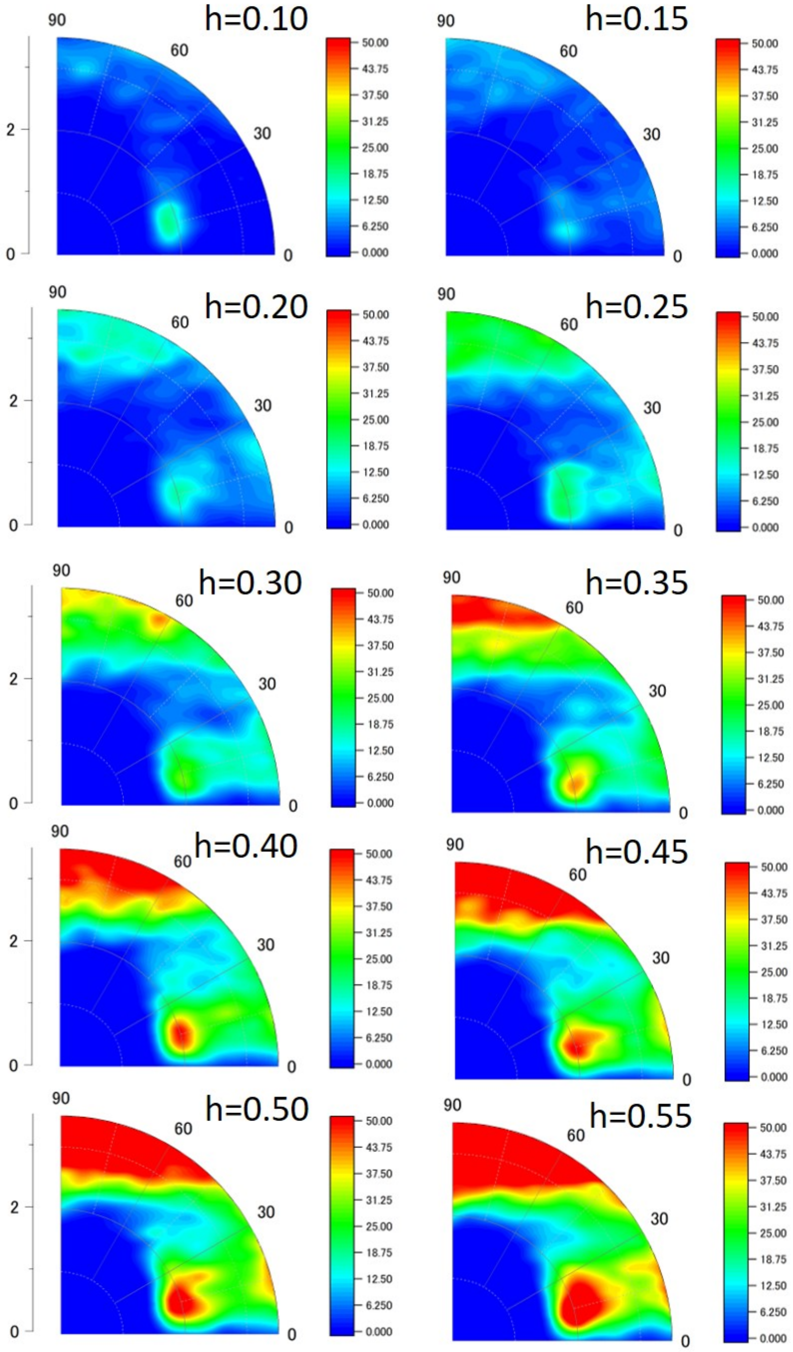

Continued

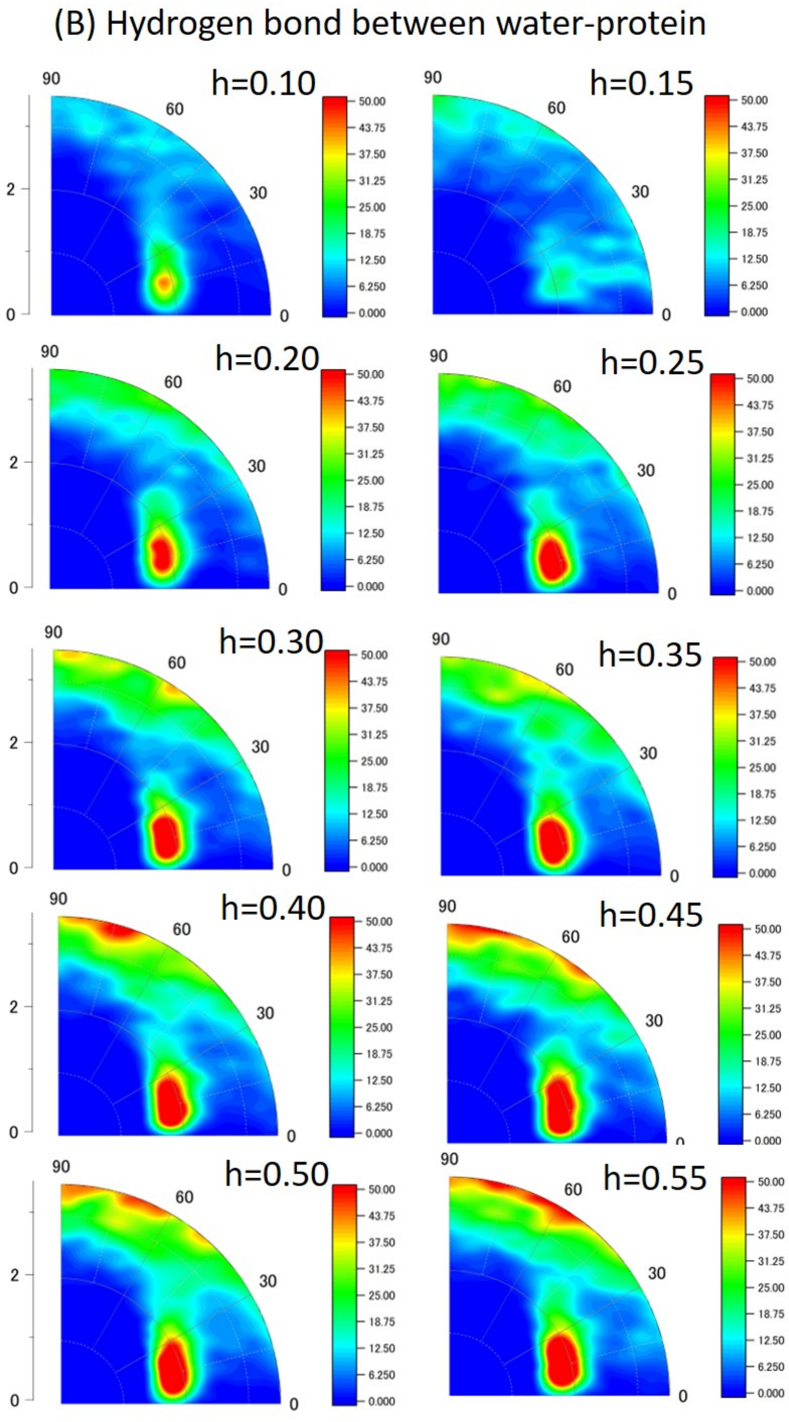

Supplementary Figure S4. Geometrical distribution of O-H…O (A) between water and water and (B) water and protein at various hydration levels from h = 0.10 (g water/g protein) to h = 0.55 (g water/g protein).

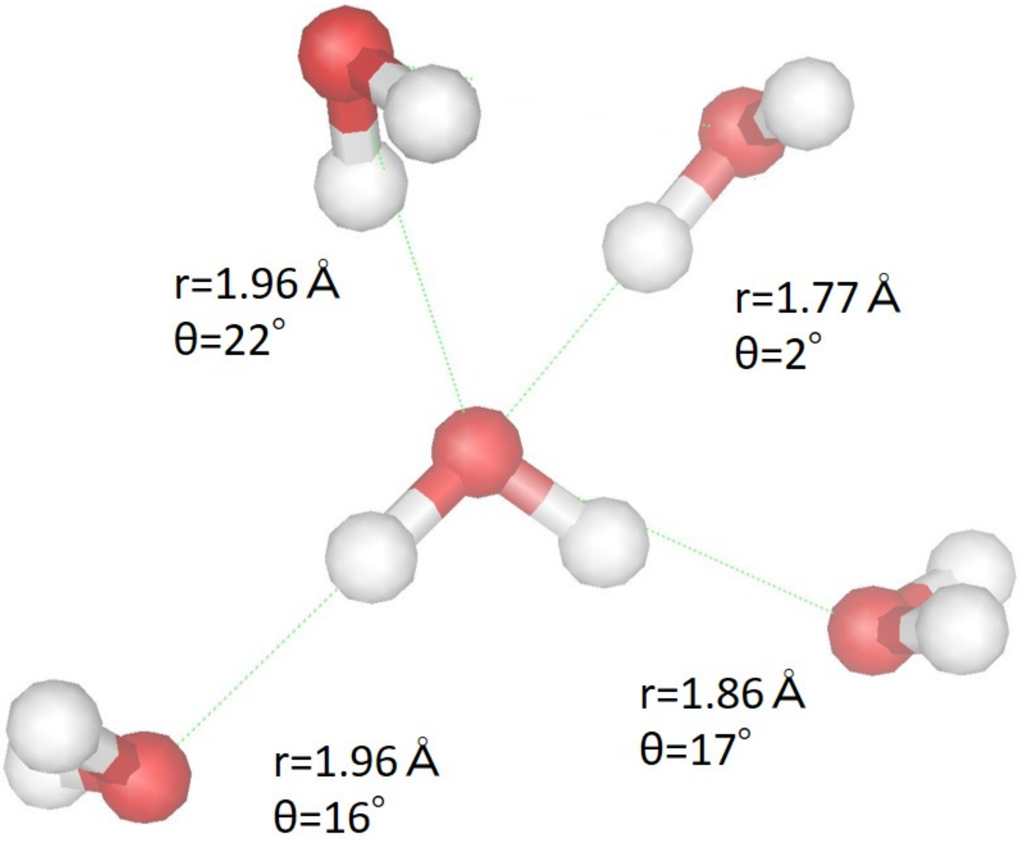

Supplementary Figure S5. Typical tetrahedral hydrogen bond structure of bulk water. Green dotted lines indicate the hydrogen bonds between water molecules. Distances and angles of the hydrogen bonds are shown.

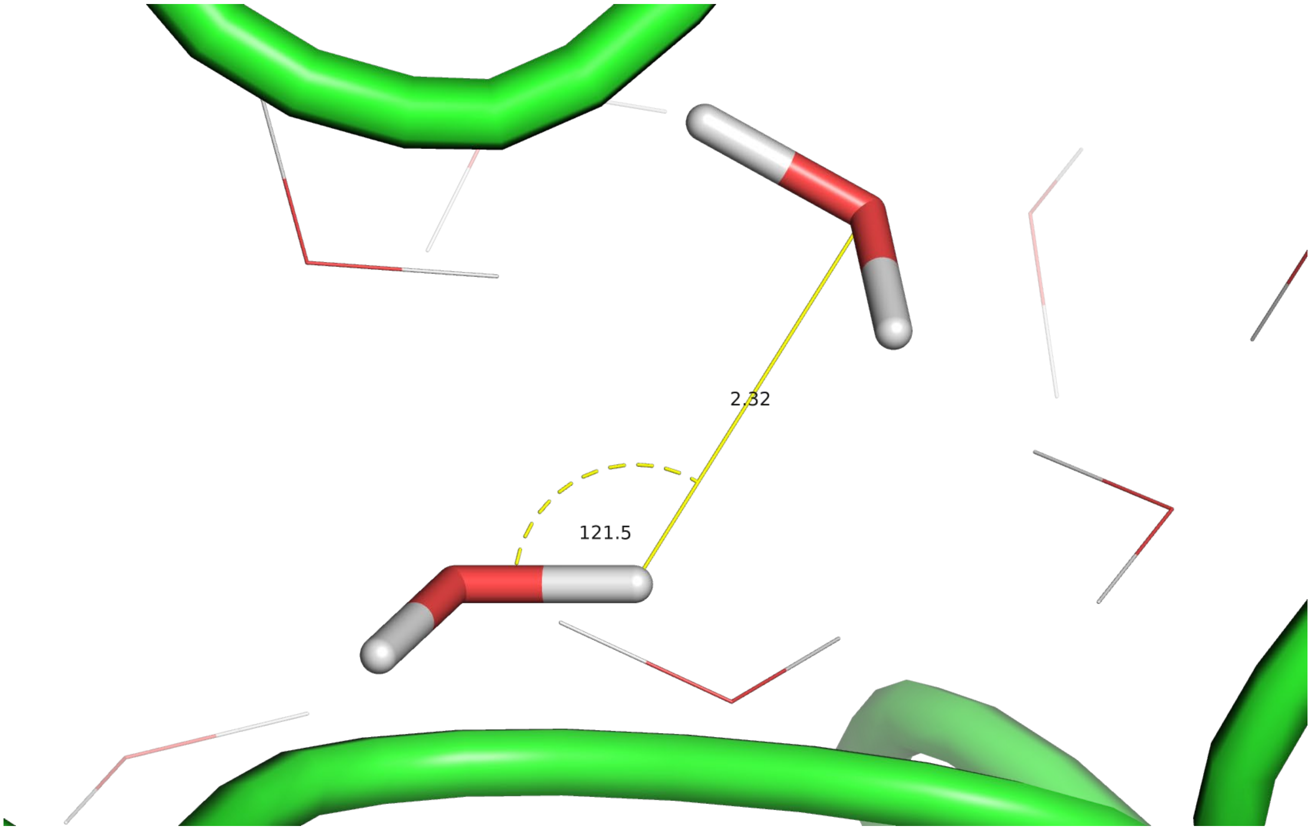

Supplementary Figure S6. A typical case of the relative geometry of two water molecules observed at r = 2.5 Å and $\theta = 60^{\circ}$ (PDB code: 3HGN). In this geometry, a hydrogen bond between two water molecules can be formed.
